# National health inequality monitoring: current challenges and opportunities

**DOI:** 10.1080/16549716.2017.1392216

**Published:** 2018-02-20

**Authors:** Ahmad Reza Hosseinpoor, Nicole Bergen, Anne Schlotheuber, Ties Boerma

**Affiliations:** ^a^ Department of Information, Evidence and Research, World Health Organization, Geneva, Switzerland; ^b^ Faculty of Health Sciences, University of Ottawa, Ottawa, Canada; ^c^ Department of Community Health Sciences, University of Manitoba, Winnipeg, Canada

**Keywords:** Monitoring Health Inequality in Indonesia, Health equity, health inequality, monitoring, Sustainable Development Goals, health information systems

## Abstract

National health inequality monitoring needs considerably more investment to realize equity-oriented health improvements in countries, including advancement towards the Sustainable Development Goals. Following an overview of national health inequality monitoring and the associated resource requirements, we highlight challenges that countries may encounter when setting up, expanding or strengthening national health inequality monitoring systems, and discuss opportunities and key initiatives that aim to address these challenges. We provide specific proposals on what is needed to ensure that national health inequality monitoring systems are harnessed to guide the reduction of health inequalities.

## Background

National health inequality monitoring aims to provide the evidence for policies, programmes and practices that tackle health inequities. It shows how various subgroups within a country are performing with regard to health, and permits comparisons between subgroups; further, it demonstrates how a country is progressing towards its equity goals and targets, and whether pathways towards universal health coverage exacerbate or reduce inequalities. While global monitoring of inequalities between countries based on national averages is an important endeavour (and traditionally, the predominant form of global monitoring), this article focuses on health inequality monitoring systems within countries, acknowledging that global comparisons of within-country inequalities enables benchmarking and more nuanced analyses.

Inputs from health inequality monitoring are a critical component of health situation analyses, required to inform priority setting [1]. By fully integrating health inequality monitoring into national health information systems, countries can produce regular data on all disadvantaged populations for all relevant indicators [1,2]. These data then can be used to design and deliver population health interventions that are responsive to greater needs in certain subgroups, where appropriate. In the context of the Sustainable Development Goals, health inequality monitoring is gaining attention as a political priority, alongside advances in the theoretical and technical underpinnings of monitoring (see Box 1. Monitoring health inequalities in the context of the Sustainable Development Goals).

**Box 1.** Monitoring health inequalities in the context of the Sustainable Development Goals.

The United Nations 2030 Agenda for Sustainable Development provides major impetus for establishing and/or strengthening health inequality monitoring systems [3]. Leaving no one behind is a central theme of the declaration, which was signed by all United Nations Member States. Reducing inequality within and among countries is one of the 17 Sustainable Development Goals (SDGs) (SDG 10) and is central to other goals such as ending poverty (SDG 1), ending hunger (SDG 2), ensuring inclusive and equitable quality education (SDG 4) and achieving gender equality (SDG 5) [4]. The health-related goal (SDG 3) calls upon countries to ‘ensure healthy lives and promote well-being for all at all ages’, with universal health coverage as the target that underpins all other health and health-related targets (SDG target 3.8) [5]. Health inequality monitoring is vital for tracking progress towards universal health coverage to ensure that disadvantaged populations achieve accelerated gains alongside overall improvement in the broader population, thus narrowing coverage gaps [6,7]. It is also important for countries to track progress towards national goals or global goals (if considered relevant in the national context). Global monitoring of SDGs with an equity lens is currently underway [8,9].

## What does national health inequality monitoring entail?

**Figure 1.**
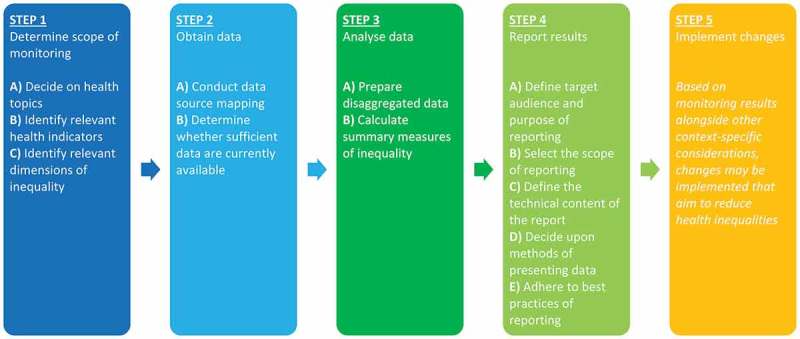
Health inequality monitoring flowchart. Source: National health inequality monitoring: a step-by-step manual [11].

Health inequality monitoring tracks the observed differences in health between population subgroups. It consists of data collection, analysis, interpretation and communication. The process of health inequality monitoring, detailed in Figure 1, begins with the selection of relevant health indicators and inequality dimensions, followed by obtaining data, analysing data, reporting results and implementing changes based on these results. The process then repeats itself, as new changes (e.g. to programmes, policies and practices) necessitate ongoing monitoring [10]. Technical information and considerations pertaining to the steps of health inequality monitoring have been published previously [11]. National health inequality monitoring systems should strive to cover diverse health topics and multiple components of the health sector [11–13].

The requirements of national health inequality monitoring systems include: high-quality, relevant data about a range of health indicators and dimensions of inequality; technical knowledge and resources to perform analyses; and capacity to interpret results, communicate them effectively, and advocate for/implement change, as needed [11]. To meet these requirements, national health inequality monitoring systems must be supported politically, financially and by adequate human resources. With sufficient investment and commitment, national health inequality monitoring systems can be established and strengthened progressively, incrementally improving upon the range and quality of data, as well as building technical capacities as an integral part of country health information systems [2,10].

## Key challenges

There is scope for improvement in health inequality monitoring in virtually all countries. High-income countries often have sophisticated health information systems, but their capacity for monitoring health inequality varies. For example, Campos-Matos et al. point to a ‘real or perceived lack of evidence on health inequalities’ in Portugal as a barrier to taking policy action to address health inequalities [14]. The UK has regular and systematic reporting of national health inequality monitoring, whereas the USA does not [15]. Low- and middle-income countries often do not have sufficient integration of health inequality monitoring into their health information systems; some middle-income countries, however, have institutionalized regular health inequality monitoring in some health topics. Mexico, for instance, has institutionalized monitoring systems – which capture health inequality monitoring – that are linked to public policies that target the poor [16]. Brazil collects municipality-level data and conducts household surveys, which enable extensive national health inequality monitoring [17,18].

A first set of challenges is related to data collection [10,19]. Countries may lack strong data collection practices (that is, data availability and quality may be poor), and/or data collection may not be equity-oriented (that is, data cannot be disaggregated by dimensions of inequality). For many countries, the situation may be mixed: high-quality data sources that are equity-oriented may exist for a limited number of health topics, but be lacking for other health topics [8,20].

Population-based health surveys – generally conducted at the household level – can be powerful instruments for health inequality monitoring, given that they typically collect data about multiple aspects of health and for diverse dimensions of inequality. In many countries they are the predominant data source for health inequality monitoring, especially on coverage of health interventions and risk factors. Certain challenges arise, however, when relying on population-based surveys for health inequality monitoring. Surveys do not cover certain important health topics such as noncommunicable diseases. Many countries conduct surveys infrequently, resulting in major gaps. Population surveys may not include certain population subgroups, such as small minorities, special populations (e.g. prisoners) or individuals that are hard to identify (e.g. injection-drug users). Special, targeted data collection efforts may be required.

Routine reports from health facility can also be an important source of information, especially if there are reliable estimates of the size of the target population. The most important application is the analysis of inequalities between geographic and administrative areas. Countries with paper-based data collection practices generally have fewer possibilities for health inequality monitoring than those with electronic systems, as the collection and analysis of disaggregated data is limited in paper-based systems.

A second set of challenges relates to the analysis stage of national health inequality monitoring. Navigating the technical complexities of health inequality monitoring requires expertise and dedicated training [21]. In some cases, technical knowledge within the country may be lacking within Ministries of Health and in statistical or research institutions. In other instances, those with advanced technical knowledge for measuring health inequalities (e.g. in universities or technical institutions) do not have access to all the data or resources to conduct health inequality monitoring at a national level.

Third, countries may lack capacity to effectively report and communicate the results of national health inequality monitoring. Effective communication to different target audiences requires a specialized set of skills that are distinct from the technical skills needed to do data collection or analysis [8,20,22]. Further, without investment in developing context appropriate reporting channels – such as printed reports, online portals, databases and others – the results of monitoring may not reach the target audience.

A final set of challenges relates to implementing the results of national health inequality monitoring. Countries may lack the planning and coordination across levels and sectors of governance to successfully tackle the root causes of health inequalities [23]. In some settings, other barriers to implementing change may also be at play, such as: a lack of political will or incentives; absence of a legal framework to mandate action; paucity of an evidence-basis for how to address inequalities; and covert ideologies that are discriminatory towards vulnerable populations.

## Opportunities and key initiatives

Since health inequalities occur everywhere, all countries stand to benefit from strengthening health inequality monitoring systems. Overcoming the challenges requires robust systems and infrastructure that are run by strong national institutions. It also requires dedicated efforts to build and maintain the knowledge, technical skills and capacity to conduct monitoring, and may require systemic changes to institutions and legal frameworks. For some countries, developing these resources may be a long-term and incremental endeavour, while for others, existing resources may be strengthened and fine-tuned.

National health information systems are based on multiple data sources including civil registration and vital statistics (CRVS), censuses, population-based surveys, routine facility information, public health/disease surveillance, administrative data, and non-health sector information [1,10]. All data sources should include relevant dimensions of inequality, so that disaggregation is possible. Where applicable, the health data should be able to be linked to other types of data through common individual identifiers; linking of health data and dimension of inequality is also possible at the aggregate level, such as for small geographic areas. The use of area-based units confers practical advantages related to understanding the results of health inequality monitoring and implementing equity-oriented changes [24].

A number of resources and initiatives support countries in developing and scaling up national data collection infrastructure. For instance, the Global Financing Facility supports strengthening CRVS in low- and middle-income countries [25], in line with national priorities and strategic plans developed by the World Bank Group and the WHO [26]. In countries where health information systems lack data sources for health inequality monitoring, population-based health surveys are a common source of data. Operating across many countries, initiatives such as the USAID-funded Demographic and Health Surveys and UNICEF-funded Multiple Indicator Cluster Surveys (MICS) are nationally representative surveys that consist of modules focusing on specific health topics [27–29].

Several international initiatives and organizations offer support in building capacity for data analysis. For instance, the Countdown to 2030 initiative (previously Countdown to 2015) collaborates with regional and country institutions to promote better measurement and monitoring in topics related to reproductive, maternal, newborn, child and adolescent health. Countdown focuses on strengthening regional and country capacity for evidence generation and use, including forging collaborations to foster country-based analysis and reporting on progress on aspects of the 2030 Agenda for Sustainable Development [30]. The WHO has provided capacity building workshops at national and regional levels, teaching participants to perform and interpret health inequality analyses, and providing opportunities for networking among participants. In addition, a training of trainers component prepares participants to teach these skills in subsequent workshops [31]. As part of its MICS programme, UNICEF supports countries in improving their capacity in data analysis, dissemination and use through regional workshops and in-country technical assistance [32]. The Health Data Collaborative, a multi-partner platform to strengthen country health information systems, has established a working group to develop core set of tools and methods to enhance the analysis of health data [33].

Communication and reporting about health inequalities should be done regularly, and the results of health inequality monitoring should be integrated into key Ministry of Health reports, such as health sector progress and performance reports, and annual health statistical reports. Through the WHO, tools are available to assist with health inequality analysis and reporting. Statistical codes in R, Stata, SAS and SPSS facilitate the calculation of disaggregated estimates from household survey data [34]. The Health Equity Assessment Toolkit (HEAT) software package facilitates the calculation of summary measures of inequality, drawing from an existing database of disaggregated data; HEAT Plus, the upload-database edition of the software, additionally allows users to import their own database. Additionally, HEAT and HEAT Plus have the capability to present data in an interactive way and generate tables and graphs that can be downloaded for reporting purposes [35].

Determining how to effectively implement changes based on the results of health inequality monitoring is a growing area of interest in research and practice. One strategic entry point is through national health plans, which detail how a country allocates its budget for health, and guide programme planning and implementation [36]. The WHO has published an eight-step approach (the *Innov8 approach*), which helps countries to systematically and comprehensively orient the delivery and design of national health programmes for the reduction of health inequalities. This approach draws on the strengths of multidisciplinary teams, exploring the underlying causes of inequities and encouraging sustainable change through improved governance and accountability [23]. Advocacy efforts, which draw on a diversity of actors from civil society, research and policy environments, also play a role in bringing about action on health inequalities [37].

## Conclusion

Establishing and strengthening national health inequality monitoring systems is an essential investment as countries move forward to ensure that policies, programmes and practices are equity-oriented and effective. Countries, however, face common types of bottlenecks that restrict the extent to which health inequality monitoring can be done, and impede the ability to implement changes. These barriers emerge from limitations associated with health information systems, but also from political, financial, social and cultural influences. Key opportunities that countries should move forward on include: building robust data collection infrastructure, supported by national institutions; building knowledge and technical capacity for equity analysis and communication; and determining effective ways to use the results of health inequality monitoring for better resource allocation and programme implementation, including identifying and addressing barriers to action.

## Paper context

Integrating health inequality monitoring into national health information systems is paramount to ensure that health policies and programmes are equity-oriented (i.e. achieve early and accelerated improvement in disadvantaged populations). This paper characterizes four common types of challenges that countries face related to data collection, analysis, communication/reporting and implementing results, and identifies strategies and resources for advancement. Strategic investments to overcome challenges can help to strengthen and expand health inequality monitoring opportunities.
